# A multi-omics study to investigate the progression of the Correa pathway in gastric mucosa in the context of cirrhosis

**DOI:** 10.1186/s13099-023-00571-y

**Published:** 2023-09-26

**Authors:** Ruiguang Ma, Qian Li, Guoxian Yu, Jun Wang, Yueyue Li, Xinyan Xu, Yiqing Zhu, Min Dong, Yanjing Gao, Lixiang Li, Zhen Li

**Affiliations:** 1 Department of Gastroenterology, Qilu Hospital of Shandong University, No. 107, Wenhuaxi Road, Jinan, 250012 China; 2 School of Software, Shandong University, Jinan, China; 3 SDU-NTU Joint Centre for AI Research, Shandong University, Jinan, China

**Keywords:** Cirrhosis, Premalignant gastric lesions, Multi‐omics integration, Serum bile acids, Ostα–Ostβ

## Abstract

**Background:**

Patients with liver cirrhosis (LC) are prone to gastric mucosa damage. We investigated the alterations of gastric mucosa in LC patients and their possible mechanisms through multi-omics.

**Results:**

We observed significant gastric mucosa microbial dysbiosis in LC subjects. Gastric mucosal microbiomes of LC patients contained a higher relative abundance of *Streptococcus*, *Neisseria*, *Prevotella*, *Veillonella*, and *Porphyromonas*, as well as a decreased abundance in *Helicobacter* and *Achromobacter*, than control subjects. The LC patients had higher levels of bile acids (BAs) and long-chain acylcarnitines (long-chain ACs) in serum. The gastric mucosal microbiomes were associated with serum levels of BAs and long-chain ACs. Transcriptome analyses of gastric mucosa revealed an upregulation of endothelial cell specific molecule 1, serpin family E member 1, mucin 2, caudal type homeobox 2, retinol binding protein 2, and defensin alpha 5 in LC group. Besides, the bile secretion signaling pathway was significantly upregulated in the LC group.

**Conclusions:**

The alterations in the gastric mucosal microbiome and transcriptome of LC patients were identified. The impaired energy metabolism in gastric mucosal cells and bile acids might aggravate the inflammation of gastric mucosa and even exacerbate the Correa’s cascade process. The gastric mucosal cells might reduce bile acid toxicity by bile acid efflux and detoxification.

*Trial registration*: ChiCTR2100051070.

**Supplementary Information:**

The online version contains supplementary material available at 10.1186/s13099-023-00571-y.

## Background

Liver cirrhosis (LC) is a common chronic progressive liver disease, the 11th most common cause of death worldwide. About 1 million deaths annually are attributable to liver cirrhosis [1]. The liver interacts directly with the gut through the hepatic portal and bile secretion systems [2]. Increasing studies indicate that patients with cirrhosis have altered gut microbiota, which is one of the significant factors in promoting the progression of liver cirrhosis [3].

Changes in microbiota with cirrhosis have been demonstrated in the stool, intestinal mucosa, serum, ascites, and saliva samples, pointing toward a global mucosal immune impairment in patients with liver cirrhosis [4]. Among published studies, some results indicated that patients with liver cirrhosis and portal hypertension had mucosal abnormalities. They include mucosal inflammatory-like abnormalities (edema, erythema, granularity, and friability), erosive gastritis, gastric ulcer, and intestinal metaplasia [5–7]. Enrichment of certain pathogenic bacteria in the stomach can promote further progression of gastric inflammation or gastric cancer [8]. However, the characterization of gastric microbiota in cirrhosis and their relationship with gastric mucosal abnormalities is unclear.

In addition, patients with cirrhosis also have alterations in serum metabolite [9], which could collaborate with mucosal microbiota to irritate lesions. Technological advances in liquid chromatography coupled with high-resolution mass spectrometry are beginning to revolutionize our understanding of the cause of liver cirrhosis. Recent advances indicate that a general metabolic alteration is a hallmark of the intricate road to decompensated cirrhosis [10]. This metabolic alteration is characterized by decreased β-oxidation of fatty acids in the mitochondria and a concomitant increase in extramitochondrial glucose utilization via glycolysis [10, 11]. Nevertheless, the interaction between gastric flora and blood metabolism in patients with liver cirrhosis is still unknown. It is also unclear whether gastric mucosal dysbiosis in cirrhosis patients has any underlying relationship with serum metabolite.

We hypothesized that there are significant alterations in the gastric mucosal microbiota in cirrhosis, like the gut, which may exacerbate mucosal abnormalities in the stomach. We also postulated that perturbations of the gastric mucosal microbiota have significant correlations with serum metabolite in patients with liver cirrhosis, and poses significant effects on the development of the disease. To answer these questions, we performed 16S rRNA high-throughput sequencing to characterize the gastric mucosal microbial communities and liquid chromatography–mass spectrometry (LC–MS) to assess serum metabolism in the same group of patients with liver cirrhosis.

## Methods

### Subjects and sample collection

We performed an observational study of 64 patients with liver cirrhosis and 59 non-liver cirrhosis patients. Patients were recruited at the Qilu Hospital of Shandong University from September 2021 to April 2022. According to international guidelines, LC is diagnosed based on clinical symptoms, physical signs, radiologic examination, laboratory tests, medical history, and cirrhosis-associated complications in chronic liver disease [12]. Participants aged above 18 years who signed informed consent were included into the study. The exclusion criteria were as follows: (1) cardiovascular disease, diabetes, inflammatory bowel disease, and mental disease, (2) individuals who received proton pump inhibitors, antibiotics, hormones, immunosuppressants, or chemotherapy drugs within one month of enrollment, (3) patients with positive rapid urease test. Detailed demographic, clinical, and etiology of cirrhosis model for end-stage liver disease scores were collected for all patients at the time of inclusion (Tables 1, 2). The study was registered in Chinese Clinical Trials.gov (http://www.chictr.org.cn/index.aspx, ChiCTR2100051070). The study was approved by the Ethics Committee of Qilu Hospital of Shandong University and complied with the Declaration of Helsinki.

**Table 1:** Demographic characteristics of the groups

Omics type	Characteristic	Patients with cirrhosis	Control subjects	P value
Gastric microbiota genomic	Number	39	30	–
	Age, mean ± SD, years	53.40 ± 10.8	48.20 ± 14.6	0.09
	Male/female	24/15	15/15	0.46
	BMI, mean ± SD, kg/m^2^	24.02 ± 3.25	23.28 ± 14.62	0.36
	MELD scores	7.42 ± 4.39	–	–
Serum metabolomic	Number	22	44	–
	Age, mean ± SD, years	53.04 ± 11.08	48.32 ± 14.37	0.15
	Male/female	15/8	25/19	0.51
	BMI, mean ± SD, kg/m^2^	24.30 ± 4.07	22.64 ± 1.83	0.08
	MELD scores	7.70 ± 3.86	–	–
Gastric mucosa transcriptomic	Number	10	10	–
	Age, mean ± SD, years	58.10 ± 11.49	57.20 ± 14.92	0.88
	Male/female	8/2	3/7	0.07
	BMI, mean ± SD, kg/m^2^	24.51 ± 2.59	24.80 ± 3.07	0.82
	MELD scores	9.60 ± 4.60	–	–

Comparisons between patients with cirrhosis and controls

*BMI* body mass index, *MELD* model for end-stage liver disease, *SD* standard deviation

**Table 2:** Etiology of cirrhotic patients in each group

Etiology of liver cirrhosis	Gastric microbiota genomic	Serum metabolomic	Gastric mucosa transcriptomic
	n = 39	n = 22	n = 10
Hepatitis B virus	21	14	4
Hepatitis C virus	2	0	0
Alcohol-related liver disease	3	1	3
Autoimmune hepatitis	5	4	0
Primary biliary cholangitis	4	2	0
Budd-chiari syndrome	0	0	1
Nonalcoholic steatohepatitis	1	1	0
Cryptogenic cirrhosis	3	0	2

We performed high-definition gastroscopy (Pentax EG29-i10, Pentax, Tokyo, Japan) for all enrolled participants. Each piece of mucosa from the gastric antrum to body of the stomach was examined by gastroscopy, and a rapid urease test was carried out. Two pieces of gastric tissue fragments with negative rapid urease test were collected using 2.2 mm sterile biopsy forceps, and all were stored immediately at − 80 °C for the subsequent procedure. One piece was used for 16S rRNA sequencing, and the other was stored in RNALater according to the manufacturer’s instructions and stored at − 80 ℃ for subsequent transcriptomics analysis. All examinations were performed by two experienced gastroenterologists with at least five years’ experience (YYL, YJG).

The blood samples were drawn by experienced nurses after 8h fasting on the 2nd day morning after admission, and were collected in 10 mL EDTA tubes. Samples were centrifuged at 13,000*g* for 5 min at 4 ℃ within one hour after collection, and the plasma was frozen at − 80 °C freezer. Standard laboratory parameters were evaluated by the central laboratory of the Qilu Hospital of Shandong University (Additional file 1: Table S1). Blood samples, and gastric mucosa were transported under dry ice to laboratory, where they were thawed at the time of analysis. The flow chart of the study population is shown in Additional file 2: Figure S1A.

### Microbiome analysis

We performed microbiota analysis using alpha and beta diversity analyses. Samples with < 1% *Helicobacter pylori* relative abundance were grouped as *H. pylori*-negative, while samples with > 1% *H. pylori* relative abundance were grouped as *H. pylori*-positive [13, 14]. Differentially abundant bacterial taxa are identified by the linear discriminant analysis (LDA) effect size (LEfSe) method. We used microbial network analysis to identify clusters of microbial taxa that were highly correlated (correlation coefficients < -0.5 or > 0.5, q < 0.05).

### Metabolome analysis

Unsupervised principal component analysis and partial least squares discriminate analysis (PLS-DA) was used to assess the global metabolic alterations between groups. The partial least squares discriminate analysis model was used with the first principal component of variable importance in projection values (VIP > 1.0) combined with Student’s t-test (*P* < 0.001) to determine the significantly different metabolites between the LC group and control group. The differentially accumulated metabolites were mapped to the Kyoto Encyclopedia of Genes and Genomes database (KEGG) for descriptive annotation. Spearman method was used to analyze the correlation coefficients for the metabolome and microbiome data integration.

### Transcriptome analysis

We identified genes with false discovery rate (FDR) < 0.05 and |log2FC|> 1 in a comparison as significant differential expression gene (DEG). DEGs were considered significantly enriched in a KEGG pathway at *q* ≤ 0.05 compared with the whole transcriptome background.

### Statistical analysis

Statistical analysis was performed using the Mann–Whitney U test and one-way ANOVA when appropriate. Statistical significance was taken as *P* < 0.05. Data were analyzed using SPSS software version 25.0. All authors had access to the study data and had reviewed and approved the final manuscript.

## Results

### Increased alpha-diversity and altered overall microbial composition in LC

**Fig. 1:**
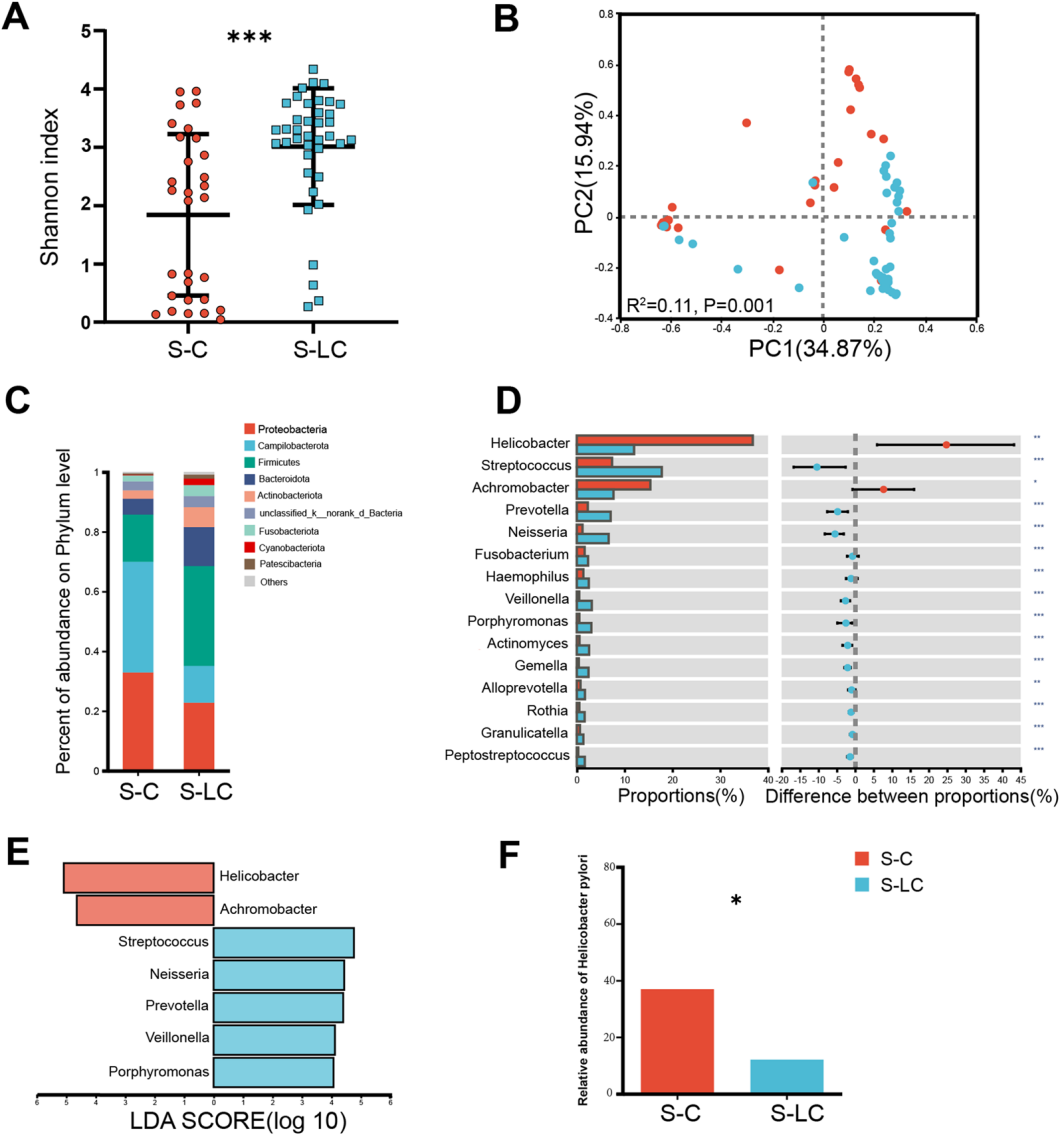
Gastric mucosal microbiome dysbiosis between S-C and S-LC. S-C represents gastric mucosa derived from the control group, and S-LC represents gastric mucosa derived from the liver cirrhosis group. In **A**, **B**, **D**–**F**, red is the S-C group, and blue is the S-LC group, *p < 0.05, **p < 0.01, ***p < 0.001. **A** Increased microbial richness, estimated by Shannon index. **B** Gastric mucosal microbiota showed relative clustering between control subjects compared with all patients with cirrhosis. **C** Relative abundance of microbial species at the phylum level. **D** The difference in species composition at the genus level. **E** The LDA value distribution histogram shows the species between S-C and S-LC at the genus level (LDA > 4). **F** The relative abundance of *H. pylori* was higher in S-C than in S-LC. Statistical significance was determined by the Mann–Whitney U test

Total of 69 subjects, including 30 control subjects (S-C) and 39 patients with cirrhosis (S-LC), with similar demographics, were included in this study and the information were shown in Tables 1 and 2. We compared the alpha diversity of gastric microbiota between the S-LC and S-C group, and the Sobs (*P* < 0.05), Shannon (*P* < 0.001), Ace (*P* < 0.05), and Chao1 (*P* < 0.05) values were significantly higher in S-LC (Additional file 1: Table S2, Fig. 1A). Meanwhile, beta diversity analysis showed separate clusters for S-LC and S-C (*P* = 0.001, Fig. 1B). The gastric microbiota was dominated by eight phyla: Proteobacteria, Campilobacterota, Firmicutes, Bacteroidetes, Actinobacteria, unclassified_k__norank_d__Bacteria, Fusobacteria, and Cyanobacteria (Fig. 1C), although the two groups presented in different order of relative abundance at the phylum level. The gastric microbiota in liver cirrhosis had an over-representation of Firmicutes, Bacteroidetes, Actinobacteria, and Fusobacteriota (*P* < 0.001; Additional file 3: Figure S2). At the genus level, several genera, including *Streptococcus*, *Prevotella*, *Neisseria*, *Fusobacterium*, *Haemophilus*, *Veillonella*, *Porphyromonas*, *Actinomyces*, *Gemella*, *Alloprevotella*, *Rothia*, *Granulicatella*, and *Peptostreptococcus*, significantly increased in relative abundance between the S-C and S-LC (Fig. 1D). The *Helicobacter* and *Achromobacter* decreased in LC (Fig. 1D).

### Bacteria differentially abundant in LC versus controls

We performed linear discriminant analysis effect size at the genus level to further identify the gastric-specific species signatures. Seven bacterial taxa showed distinct relative abundances between the two groups. Increased abundance in bacteria, including *Streptococcus*, *Neisseria*, *Prevotella*, *Veillonella*, and *Porphyromonas*, and decreased abundance in *Helicobacter* and *Achromobacter* were observed in S-LC (LDA score > 4, *P* < 0.05; Fig. 1E). Furthermore, we found that the *H. pylori* infection was significantly lower in S-LC than in S-C (Fig. 1F).

To understand the potential interplay among differentially abundant bacteria in S-LC and control, we performed the network topology analysis (Additional file 4: Figure S3) at the genus level. In S-C group, co-occurrence interactions were observed, reflecting synergistic interkingdom interactions’ contribution to gastric microbiota homeostasis (Additional file 4: Figure S3A). Much fewer co-occurrence interactions were observed in S-LC (Additional file 4: Figure S3B). In addition, *H. pylori* had a co-exclusive association with other gastric microbes in S-C predominantly. However, these correlations were relatively infrequent in S-LC. Collectively, the above microbial analysis indicated a state of dysbiosis in the mucosal microbiome of LC patients.

### Blood metabolism changes with LC

**Fig. 2:**
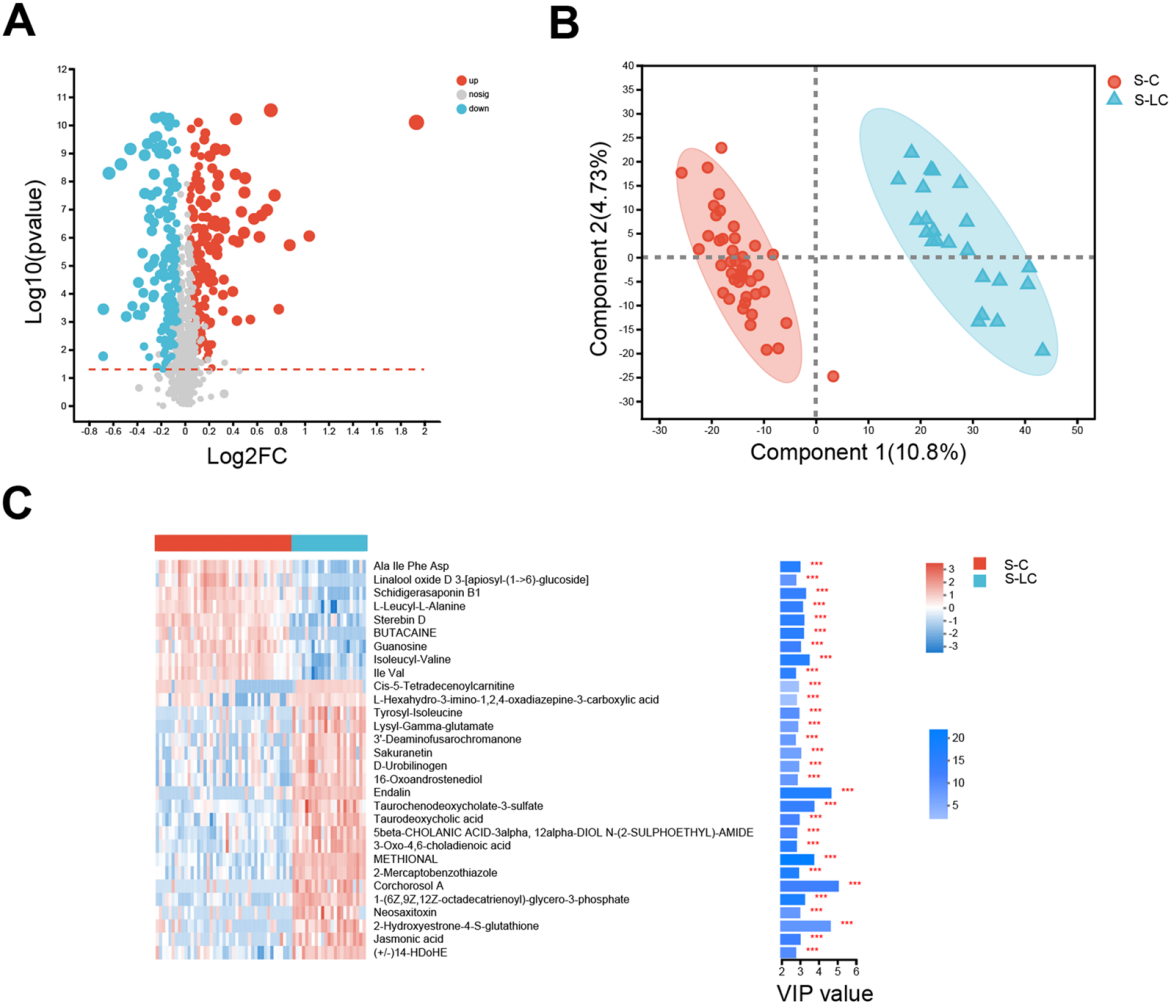
The liver cirrhosis group is associated with altered serum metabolites. **A** Differential metabolites by volcano diagram. **B** PLS-DA of the metabolites across the two groups. **C** VIP scores with the corresponding expression heatmap. On the left side is the metabolite heatmap. On the right side is the metabolite VIP bar graph. The bar length indicates the contribution of the metabolite to the difference between the two groups. The higher value means the metabolite is more difference between the two groups. The bar color indicates the P value of the metabolite between the two groups, *p < 0.05, **p < 0.01, and ***p < 0.001

We recruited 22 cirrhotic patients (age 53.04 ± 11.08 years, 15 men) and 44 control patients (age 48.32 ± 14.37 years, 25 men) who agreed to give blood serum (Tables 1, 2). We used liquid chromatography-mass spectrometry; to analyze the serum samples, and the abundance profiles were obtained for 1540 annotated serum metabolites. It was found that 492 of 1540 metabolites had significantly different abundances (Fig. 2A). We subsequently performed the partial least squares discriminate analysis and the results revealed visual separation between these groups without overfitting (Fig. 2B). In addition, PLSDA-VIP table (Fig. 2C, Additional file 1: Table S3) showed top 30 metabolites with VIP > 1.0 and *P* < 0.001. Of them, 9 were increased in the control group, and 21 were increased in the LC group. Notably, the VIP value of taurochenodeoxycholate-3-sulfate (VIP = 3.72), taurodeoxycholic acid (VIP = 2.93), and cis-5-tetradecenoylcarnitine (VIP = 2.89) were more than 2, indicating their significant contribution to the disease. Furthermore, we carried out an enrichment analysis for all differential metabolites using the KEGG. The results indicated that the metabolites in LC were mainly associated with sphingolipid metabolism, glycerophospholipid metabolism, cysteine and methionine metabolism (Additional file 5: Figure S4).

### Association of microbes and metabolites with LC

**Fig. 3:**
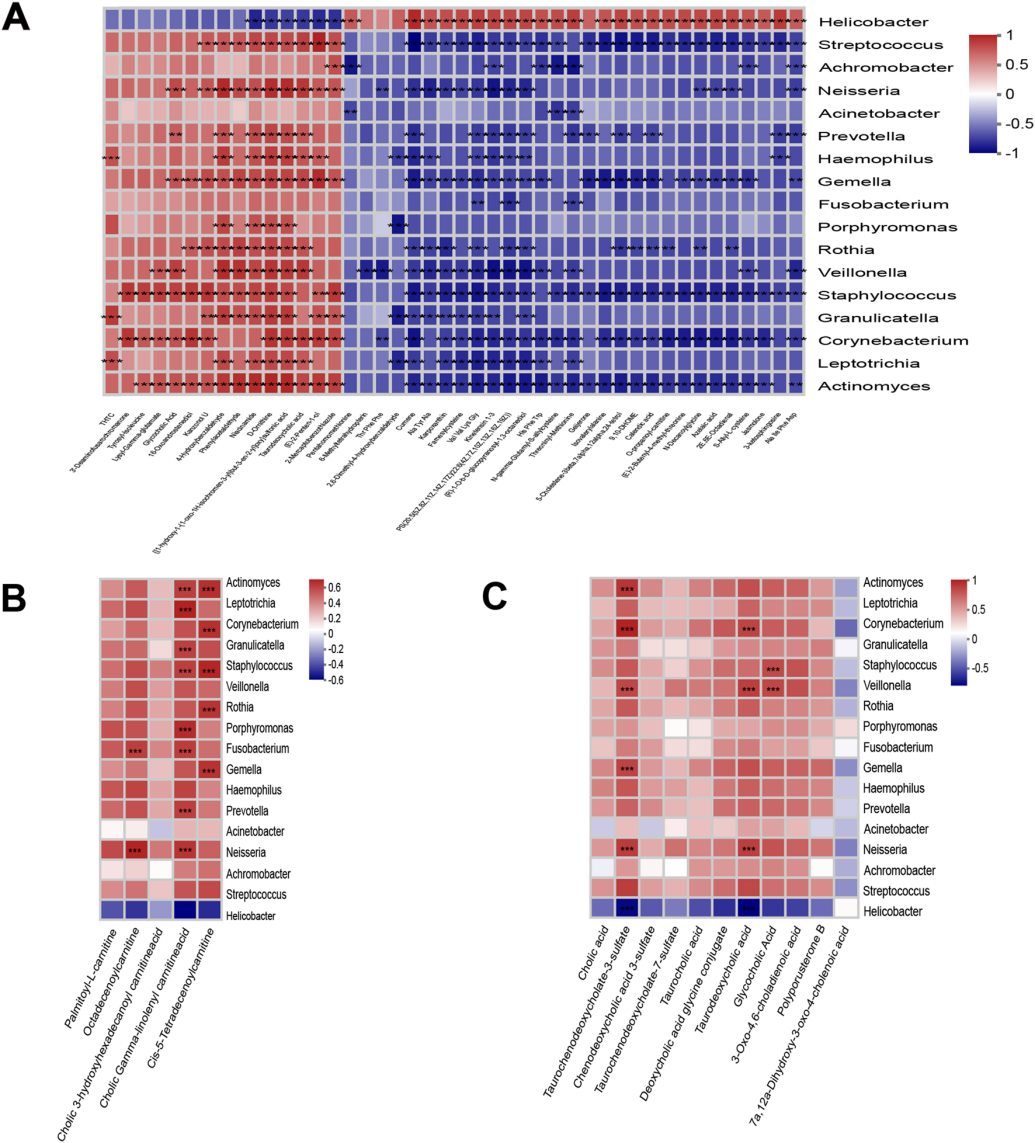
Correlation analysis between the microbiota and metabolites. **A** Heatmap of gastric mucosal flora and serum metabolite correlation. Asterisk(***) denotes a P value less than 0.05 and |r|> 0.7. **B** Heatmap of gastric mucosal flora and serum long-chain ACs correlation. Asterisk(***) denotes a P value less than 0.05 and |r|> 0.6. **C** Heatmap of gastric mucosal flora and serum bile acids correlation. Asterisk(***) denotes a P value less than 0.05 and |r|> 0.7

To further determine the relationships between gastric microbiota and metabolic changes, we subsequently perform the Spearman’s correlation analysis of serum differential metabolite and microbiota in stomach. Interestingly, there was a strong correlation between a large number of stomach microbiota and altered metabolites (Fig. 3A). The correlation was considered statistically significant with correlation coefficient |r|> 0.7 and *p* < 0.05 in this study, and results were visualized as heatmaps.

It was found that genus *Streptococcus*, enhanced in the gastric microbiota community in LC patients, was positively correlated with taurodeoxycholic acid (r = 0.71) and (E)-2-Penten-1-ol (r = 0.82). In addition, His Phe Trp and phosphatidylserine were negatively correlated with almost all the bacteria except *Helicobacter* (r = 0.65; r = 0.69). The relative concentrations of some long-chain acylcarnitines (palmitoyl-l-carnitine, octadecenoylcarnitine, 3-hydroxyhexadecanoyl carnitine, gamma-linolenic carnitine, and cis-5-tetradecenoylcarnitine) were generally seen to increase in the serum metabolites from cirrhotic patients. As shown in Fig. 3B, increased long-chain ACs were positively associated with almost all the bacteria, such as *Actinomyces* and cis-5-tetradecenoylcarnitine (r = 0.66). Besides, taurodeoxycholic acid, deoxycholic acid glycine conjugate, glycocholic acid, taurocholic acid, cholic acid, taurochenodeoxycholate-3-sulfate, taurochenodeoxycholate-7-sulfate, chenodeoxycholic acid 3-sulfate, and their derivatives were positively correlated with almost all the bacteria, except 7a,12a-dihydroxy-3-oxo-4-cholenoic acid (Fig. 3C).

### Gastric mucosal transcriptome changes in LC

**Fig. 4:**
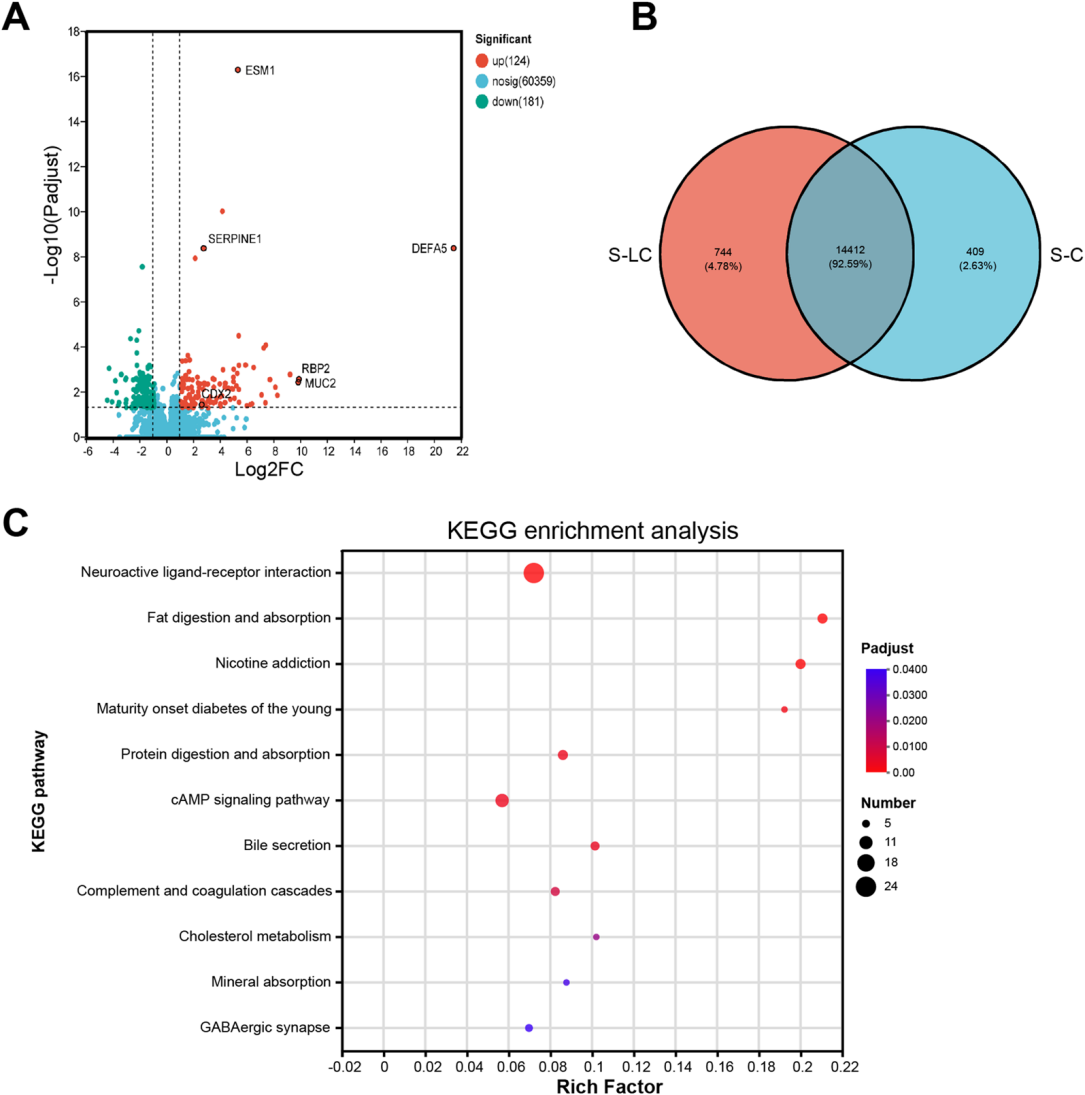
S-C represents gastric mucosa derived from the control group, and S-LC represents gastric mucosa derived from the treatment group. **A** Volcano map of DEGs. **B** Venn graph of DEGs. **C** DEGs enriched in the KEGG pathway. The X-axis represents the rich factor, indicating the ratio of enriched genes to total genes in this pathway. A more prominent rich factor indicates more significant enrichment. *ESM1* endothelial cell specific molecule 1, *SERPINE1* serpin family E member 1, *MUC2* mucin 2, *CDX2* caudal type homeobox 2, *RBP2* retinol binding protein 2, *DEFA5* defensin alpha 5, *DEG* differential expression gene

To obtain a comprehensive view of gastric mucosa influenced by microbial colonization and metabolic alterations, we further investigated the gastric mucosal transcriptome in two groups comprising 10 control and 10 LC patients. It was found that 181 and 124 genes were differentially down and upregulated, respectively. Of them, the endothelial cell specific molecule 1, serpin family E member 1, mucin 2, caudal type homeobox 2, and retinol binding protein 2 were significantly upregulated genes (Fig. 4A, B) and were associated with intestinal metastasis and gastric carcinogenesis [15–18]. We also found that defensin alpha 5 (DEFA5), a gene encoding an antimicrobial peptide, is significantly upregulated (*P* < 0.001). Then, we mapped all DEGs to KEGG pathways, and the top 11 specific pathways were represented in a bubble chart (Fig. 4C). Furthermore, genes associated with the most significantly enriched pathways (q < 0.05) were shown in Additional file 6: Figure S5. Among these 11 pathways, “neuroactive ligand-receptor interaction” was the most represented pathway. Significantly, it was found that the bile secretion signaling pathway was significantly upregulated in the LC group. In the bile secretion signaling pathway, solute carrier family 51 subunit alpha (SLC51A), solute carrier family 51 subunit beta(SLC51B), cytochrome P450 3A4(CYP3A4) were up-regulated.

## Discussion

The major conclusions derived from the current studies are that altered gastric flora and elevated bile acids might aggravate the injury to the gastric mucosa and even exacerbate Correa’s cascade process. The gastric mucosal cells might reduce bile acid toxicity by bile acid efflux and detoxification. We base this conclusion on three lines of evidence. First, we demonstrated that opportunistic pathogenic bacteria colonized the stomach mucosa of patients with cirrhosis. Next, we provided metabolic evidence to support the conclusion that gastric mucosal cells had an impaired energy metabolism. Finally, the bile secretion pathway, including genes involved bile acid efflux and detoxification, was found to upregulate in the gastric mucosal cell by transcriptome analysis.

The microbiota alteration in the skin, intestinal mucosa, ascites fluid, serum, and the oral cavity have been studied before, except for the gastric mucosa. Our data demonstrate that higher bacterial diversity and increased relative abundance of multiple bacterial genera characterize S-LC microbial dysbiosis. In our study, only *Helicobacter pylori* (Hp) was detected in the *Helicobacter*; therefore, we consider the *Helicobacter* to be Hp in the following. There was a greater relative abundance of *Streptococcus *sp*.* and *Prevotella_melaninogenica*, *Neisseria *spp*.*, and *Fusobacterium_periodonticum*, with lower Hp. These significantly increased bacteria are pathogenic oral bacteria [19–21] that have the potential to elicit an inflammatory response in epithelial cells.

Interestingly, emerging findings suggest the magnitude of roles of specific oral and gastric microbiota correlated with inflammation in the development of early-stage gastric adenocarcinoma [13]. Furthermore, as the diseases develop into more severe stages, such as atrophic gastritis, intestinal metaplasia, and gastric adenocarcinoma, the dominance of Hp begins to be displaced by other bacteria, including *Streptococcus*, *Prevotella*, and other bacteria [13, 22]. Taken together, we hypothesize that bacteria in the stomach of patients with cirrhosis may originate from the oral cavity, which may induce gastric mucosal abnormalities similar to Correa’s cascade process.

Previous studies have demonstrated that BAs modulate intestinal immunity, inflammation, and tumorigenesis [23]. We found that serum BAs were predominantly conjugated, and primary BAs were elevated. BAs can alter membrane lipid composition, and increased BAs concentrations can solubilize membranes and dissociate integral membrane proteins [24]. Of note, we also found that long-chain ACs were increased and positively correlated with most gastric mucosal flora, except Hp. In the process of β-oxidation, acylcarnitine transports acyl groups (organic acids and fatty acids) from the cytoplasm to the mitochondria so that they can be broken down to produce energy for cell activities [25]. According to the Human Metabolome Database, the primary function of most long-chain acylcarnitine is to ensure long-chain fatty acid transport into the mitochondria. Blood accumulation of long-chain ACs is a marker for incomplete fatty acid oxidation. Moreau R. et al. found that acute-on-chronic liver failure was characterized by extra-mitochondrial glucose metabolism through glycolysis and depressed mitochondrial ATP-producing fatty acid β-oxidation, which may contribute to the development of organ failures [10]. The evidence above indicates impaired energy utilization in the microcirculation of cirrhotic patients who did not have other organ complications at enrollment.

We performed a small sample transcriptome analysis to validate further the alterations in gastric mucosa—upregulation of genes associated with gastric mucosa malignancy of cirrhotic patients identified in differential gene analysis. We also found that the gene encoding DEFA5 upregulates, contributing to direct antimicrobial, mucosal host defense, and immunomodulatory properties [26]. Furthermore, the over-expression of defensins in multiple types of cancer, such as colon cancer, lung cancer, and renal cell carcinomas, suggests a potential involvement of defensins in cancer development [26–28]. Another study indicates that DEFA5 produced from metaplastic Paneth cells may accelerate the initiation of Barrett’s esophagus, which is thought to be a precancerous lesion of esophageal adenocarcinoma [29]. An ex vivo animal study shows the down-regulation of the DEFA5 gene in gastric cancer cells and that DEFA5 inhibits the growth of gastric cancer cells [30]. The underlying mechanisms of DEFA5 in the initiation and progression of gastric cancer await further studies.

Based on the pathway enrichment analysis of DEGs from gastric mucosa transcriptome, we found that the bile secretion signaling pathway was significantly upregulated in the LC group. We also found that the genes encoding the organic solute transporterα–β (Ostα–Ostβ) upregulate. Ostα–Ostβ responsible for transporting bile acids across the enterocyte basolateral membrane into the portal circulation for subsequent renal excretion [31]. CYP3A4 is the major enzyme that catalyzes the hydroxylation of bile acids at various positions, converting bile acids into more hydrophilic and less toxic molecules [32]. Upregulation of these genes is an adaptive change in gastric mucosal cells in response to bile acid.

There are some limitations of the study that are important to the trial. First, this is a single-center study with a limited sample size. Second, the etiology of LC is complex and diverse, which may yield different flora microenvironments and serum metabolites. Meanwhile, this difference can reflect the generality of the study’s conclusions. Other factors, such as drinking and smoking, were not adjusted, which may impact gastric flora and damage to the gastric mucosa, but relevant antibiotic exposures have been excluded.

In conclusion, this study is the first work on integrated metabolomic, transcriptomic, and microbial analyses to identify critical metabolites and provide insight into the molecular and metabolic mechanisms underlying the alteration in gastric mucosa. Importantly, we showed that members of gastric pathogenic taxa were accumulated, which might originate from the oral cavity. The over-represented bacteria and serum bile acids co-exacerbated the damage of gastric mucosa and even accelerated the Correa’s cascade process. Our study highlights the enrichment of the bile secretion pathway in gastric mucosal cells in the context of LC, which may potentially serve as a protective cellular mechanism for preventing gastric lesions.

## Conclusions

The major conclusions are that altered gastric flora and elevated bile acids might aggravate the injury to the gastric mucosa and even exacerbate Correa’s cascade process. We also provided transcriptomic evidence to support a protective mechanism for preventing gastric mucosal cells from bile acid toxicity. Understanding how microbiome and metabolite operate in the gastric mucosa should guide the development of new therapeutics for gastric abnormalities in patients with liver cirrhosis.

### Supplementary Information

**Additional file 1: Methods details.** **Table S1.** Biochemical indices of cirrhotic patients in each group. **Table S2.** Gastric mucosa microbiome alpha diversity index. **Table S3.** PLSDA-VIP table between patients with cirrhosis and controls.

**Additional file 2: Figure S1.** S-C represents gastric mucosa derived from the control group, and S-LC represents gastric mucosa derived from the liver cirrhosis group. (**A**) Fig A is the flow chart of the study. (**B**) Validation of the PLS-DA model using permutation testing. (**C**) The difference in species composition at the specie level. *p < 0.05, **p < 0.01, and ***p < 0.001.

**Additional file 3: Figure S2.** S-C represents gastric mucosa derived from the control group, and S-LC represents gastric mucosa derived from the treatment group. Wilcoxon rank-sum test bar plot on phylum level. **p < 0.01, and ***p < 0.001. S-C, gastric mucosa in the control group; S-LC, gastric mucosa in patients with cirrhosis.

**Additional file 4: Figure S3.** Network analysis applied to the gastric mucosal microbiota genera network analysis reveals significant interactions. The size of the node is proportional to the species' abundance. Node color corresponds to phylum taxonoic classification. The edge color represents positive (green) and negative (red) correlations, and the edge thickness is equivalent to the correlation values. All Spearman correlations have |r|> 0.5, p < 0.05. Fig A and Fig B represent the control and liver cirrhosis groups, respectively.

**Additional file 5: Figure S4.** Metabolite KEGG pathway enrichment map. Each bubble in the graph represents a KEGG Pathway. The X-axis represents the relative importance of metabolites in the pathway in terms of impact value; the Y-axis represents the enrichment significance of metabolite involvement in the pathway—log10(P value). The size of the bubble represents the Impact value. The larger the bubble, the greater the importance of the pathway. KEGG, Kyoto Encyclopedia of Genes and Genomes.

**Additional file 6: Figure S5.** Cnetplot of top 11 enriched KEGG terms between control and liver cirrhosis group.

## Data Availability

All data from this work are public and have been included in this article in the main manuscript or as additional files. Additional datasets and information are available from the corresponding author under reasonable request.

## Abbreviations

LC: Liver cirrhosis
BAs: Bile acids
long-chain ACs: Long-chain acylcarnitines
LC–MS: Liquid chromatography–mass spectrometry
LDA: Linear discriminant analysis
LEfSe: Linear discriminant analysis effect size
PLS-DA: Partial least squares discriminate analysis
VIP: Variable important in projection
KEGG database: Kyoto Encyclopedia of Genes and Genomes database
FDR: False discovery rate
FC: Fold change
DEGs: Differential expression genes
S-C: Gastric mucosa in the control group
S-LC: Gastric mucosa in patients with cirrhosis
DEFA5: Defensin alpha 5
SLC51A: Solute carrier family 51 subunit alpha
SLC51B: Solute carrier family 51 subunit beta
CYP3A4: Cytochrome P450 3A4
Hp: *Helicobacter pylori*
Ostα–Ostβ: Organic solute transporterα–β
