# Supplementary material for: A multi-omics study to investigate the progression of the Correa pathway in gastric mucosa in the context of cirrhosis
Source: Gut Pathog. 2023 Sep 26;15:45. doi: 10.1186/s13099-023-00571-y (PMC10521386; doi:10.1186/s13099-023-00571-y)
Supplement: Supplementary file 1 — Additional file 1: Methods details. Table S1. Biochemical indices of cirrhotic patients in each group. Table S2. Gastric mucosa microbiome alpha diversity index. Table S3. PLSDA-VIP table between patients with cirrhosis and controls. [file 13099_2023_571_MOESM1_ESM.docx]

**Method details**

**Abbreviations**

OTU, operational taxonomic units; LC-MS, liquid chromatograph-mass spectrometer; QC, quality control; ESI, electrospray ionization; VIP, variable important in projection; KEGG database, Kyoto Encyclopedia of Genes and Genomes database; DEGs, differential expression genes.

**Microbiome profiling**

**DNA extraction and PCR amplification**

DNA of the gastric microbiota were extracted using E.Z.N.A Soil DNA Kit (Omega Bio-Tek, Inc, GA, US). The integrity of genomic DNA was checked by electrophoresis (1% agarose gel)，and DNA concentration was measured by NanoDrop 2000 UV-vis spectrophotometer (Thermo Scientific, Wilmington, USA). The 16S rRNA V3-V4 region was amplified with primer pairs 338F (5'-ACTCCTACGGGAGGCAGCAG-3') and 806R(5'-GGACTACHVGGGTWTCTAAT-3') by an ABI GeneAmp® 9700 PCR thermocycler (ABI, CA, USA). Amplifications were performed under the following temperature profiles: initial denaturation at 95℃ for 3 min, 27 cycles of 95°C for 30s (denaturation), 55°C for 30s (annealing), and 72°C for 45s (extension). A final extension step was sustained at 72℃ for 5 min and ended at 4℃. Reaction conditions were as follows: 4.0 μl 10× PCR buffer (*TransStart* FastPfu, Dalian, China), 2 μl dNTP mixture (2.5 mM), 0.8 μl forward primer (5 μm), 0.8 μl reverse primer (5 μm), 0.4 μl *TransStart* FastPfu DNA Polymerase (5U/lL, TaKaRa, Dalian, China), 10 ng of template DNA and finally ddH_2_O up to 20 μl. PCR reactions were performed in triplicate. A random selection of samples was conducted to produce the lowest cycle number in the pre-experiment, although most samples could be expanded to suitable concentrations. All amplicons were purified using the AxyPrep DNA Gel Extraction Kit（Axygen Biosciences, Union City, CA, USA), quantified on QuantiFluor-ST (Promega, USA), then pooled into equal concentrations. Finally, paired-end sequencing was carried out on the Illumina MiSeq PE300 platform (Illumina, San Diego, CA, USA).

**Bioinformatic analysis**

After sequencing, raw reads were demultiplexed, quality-filtered by fastp version 0.19.6^[1]^, and merged by FLASH version 1.2.11^[2]^. According to UPARSE version 7.0.1090, dereplicated reads were clustered into operational taxonomic units (OTUs) at 97% identity^[3, 4]^. Additionally, the taxonomy of representative OTU sequences was determined using RDP classifier version 2.13 (70% confidence cutoff) against the 16S rRNA gene database Silva (v.138)^[5]^. After that, alpha and beta diversity analyses were performed in Mothur software (Version.1.30.2)^[6]^ and QIIME software (Version.1.9.1)^[7]^. Samples with <1% *H. pylori* relative abundance were grouped as *H. pylori*-negative, while samples with >1% *H. pylori* relative abundance were grouped as *H. pylori*-positive^[8]^. Differentially abundant bacterial taxa are identified by the linear discriminant analysis effect size method. We used the networkx in Python to estimate microbial network analysis between LC and control^[9]^. Networkx was used for the visualization of significant co-occurrence and co-excluding interactions (correlation coefficients＜-0.5 or ＞0.5, q＜0.05).

**Metabolome analysis details**

**Metabolite Extraction**

To reveal metabolic phenotypes possibly involved in cirrhosis related to the gastric microbiome and liver cirrhosis, we performed metabolic profiling of plasma by liquid chromatograph-mass spectrometer (LC-MS) analyses. Plasma samples (100 μl) were accurately weighed, and metabolites were extracted using a 400 μl methanol: water (4:1, vol/vol) solution. The mixture was then allowed to settle at -20℃. Subsequently, a high throughput tissue crusher Wonbio-96c (Shanghai wanbo biotechnology co., LTD), was used for crushing the suspension at 50 Hz for 6 min. The mixture was vortexed for 30 s and ultrasound at 40 kHz for 30 min at 5℃. Then the precipitate was followed by settling for 30 min at -20℃. After centrifugation at 1,3000 g at 4°C for 15 min, the supernatant was transferred to autosampler vials for LC-MS analysis.

**Quality Control Sample**

Prior to downstream analysis, metabolomic samples were subjected to profile-level quality control. Quality control (QC) samples were prepared by pooling equal volumes of each sample from all groups. During analysis, a QC sample was inserted every 6–10 test samples to monitor repeatability.

**LC-MS/MS Analysis**

LC-MS analyses were performed using an ExionLC^TM^AD system (AB Sciex, USA) equipped with an ACQUITY UPLC BEH C18 column (100 mm × 2.1 mm i.d, 1.7 µm; Waters, Milford, USA). The conditions for LC-MS analysis included linear gradient elution of mobile phase A: Water/formic acid (0.1%, v/v) and mobile phase B: 0.1% formic acid in acetonitrile: isopropanol (1:1, v/v), with the following step gradient program: from 0 to 3 min, 95% (A): 5% (B) to 80% (A): 20% (B); from 3 to 9 min, 80% (A): 20% (B) to 5% (A): 95% (B); from 9 to 13 min, 5% (A): 95% (B) to 5% (A): 95% (B); from 13 to 13.1 min, 5% (A): 95% (B) to 95% (A): 5% (B), from 13.1 to 16 min, 95% (A): 5% (B) to 95% (A): 5% (B) for equilibrating the systems. The column was held at 40°C, and 20 μl of the sample was injected into the LC-MS/MS with a 0.4 ml/min flow rate. During the period of analysis, all these samples were stored at 4℃.

The UPLC system was coupled to a quadrupole-time-of-flight mass spectrometer (Triple TOF^TM^5600+, AB Sciex, USA) using an electrospray ionization (ESI) source, which operated in positive mode and negative mode. The optimal conditions were set as followed: scan range(500–1000m/z); source temperature: (500℃); curtain gas (30 psi); declustering potential(80V); both Ion Source GS1 and GS2(50 psi); a rolling collision energy(20­­–60V). The source voltage was 5000 V for positive and 4000V for negative ion modes.

**Data Preprocessing and Annotation**

Baseline filtration, peak identification, peak alignment, peak integration, and retention time of the raw data were performed by Progenesis QI 2.3 (Nonlinear Dynamics, Waters, USA)^[10, 11]^.After mass spectrometry analysis, we obtained a data matrix of retention time, mass-to-charge ratio, and peak intensity. The modified 80% rule was used to remove missing values. After filtering, minimum metabolite values were input, and the sum normalized each metabolic feature.

The internal standard was used for data QC (reproducibility), and features with a relative standard deviation of less than 30% in the QC samples were retained. We used log-transformed data to compare the metabolite levels to determine significant differences between the LC and control groups. Tentative identification was proposed by comparing MS and MS/MS data with reliable biochemical databases such as the human metabolome database (http://www.hmdb.ca/) and Metlin database (https://metlin.scripps.edu/). Concretely, the tolerance range for the peak annotation was configured at ± 10 ppm for m/z. For metabolites having MS/MS confirmation, only the ones with MS/MS fragments score above 30 were considered as confidently identified. Otherwise, metabolites had only tentative assignments.

**Multivariate statistical analysis**

The metabolome analysis was performed with the R package from Bioconductor on the Majorbio Cloud Platform (<https://cloud.majorbio.com>). Unsupervised principal component analysis was performed to visualize an overview of the metabolic data, general clustering, trends, and outliers. Partial least squares discriminate analysis was used to assess the global metabolic alterations between groups. The corresponding variable important in projection (VIP) values were also calculated in the PLS-DA model. *P*-values were determined with paired Student’s two-tailed t-test. The metabolites with *p* < 0.001 and VIP > 1 were considered potential biomarkers. 1500 differential peaks were selected, including 1068 peaks in ESI+ and 472 peaks in ESI-. The differentially accumulated metabolites were mapped to the Kyoto Encyclopedia of Genes and Genomes (KEGG) database (http://www. genome.jp/kegg/) for descriptive annotation. The python SciPy software package (version 1.2.0) (https://docs.scipy.org/doc/scipy/) was exploited to identify statistically significantly enriched pathways using Fisher’s exact test. Metabolite and microbiome correlation analysis was assessed using Spearman’s correlation coefficients.

**Transcriptome analysis details**

**RNA Extraction**

The gastric mucosa of ten patients from each group was used to extract RNA for the RNA sequencing. After standing still for 5 min at room temperature, the supernatants were collected after centrifugation at 12,000 rpm, 4°C for 5 min, and supernatants were collected. After adding roughly 1/5 volume of chloroform, samples were vortexed, the mixtures stood for 5 min and were centrifuged at 12,000 rpm, 4°C for 5 min. The liquid was separated into three layers: a colorless water phase, a protein phase, and a pink organic phase from top to bottom, respectively. The resulting water phase was removed by suction immediately without touching the other phase. After that, anhydrous ethanol was added and mixed well. Then, add 10 μl magnetic beads, vortexed 15 sec to mix well, and leave for 5 min at room temperature. After briefly centrifuging, the samples were set onto the magnetic rack, and the supernatant was discarded after 3 min. The 500 μl Wash Buffer was added to the microcentrifuge tube, followed by vortexing for 15 sec to mix well. The liquid was again centrifuged, left to stand, and the supernatant was discarded. After taking tubes from the magnetic stand, the cap was opened and allowed to stand at room temperature for 3 min. Subsequently, added 50–100 μl Eluent solution vortexed for 15 sec and kept still for 15 min at room temperature. The tubes were briefly centrifuged, placed on a magnetic separation rack, rested for 3 min, then transferred 45 μl of RNA solution was to a new RNase-Free centrifuge tube. Frozen liver tissues (50–100 mg) were homogenized in 1 mL Trizol reagent according to the manufacturer’s instructions (Invitrogen). Genomic DNA was removed by using DNase (Takara). The RNA integrity was checked by 1% agarose gel electrophoresis.

The RNA quality was examined using 1% gel electrophoresis and Agilent 2100 Bioanalyzer. Sequencing libraries were constructed using only high-quality RNA samples (concentration > 50 ng/μl, OD260/280 = 1.8–2.2).

**Library Preparation and Sequencing**

The mRNA fraction was isolated from total RNA using oligo (dT) magnetic beads. A fragmentation buffer was added to cleave the mRNA into a short fragment (approximately 300 bp). Subsequently, double-stranded cDNA was synthesized using a SuperScript double-stranded cDNA synthesis kit (Invitrogen, CA) with random hexamer primers (Illumina). The viscous end of the double-stranded cDNA structure was repaired using End Repair Mix, followed by the addition of an A base at the 3′ end to form the Y-form linker. The libraries were then size-selected for cDNA target fragments of 300 bp on 2% Low Range Ultra Agarose gel followed by 15 cycles of PCR amplification using Phusion DNA polymerase (NEB). After quantification by TBS380, a paired-end RNA-seq sequencing library was sequenced with the Illumina NovaSeq 6000 sequencer (2 × 150bp read length).

**Quality Control and Read Mapping**

The paired-end raw reads were trimmed and quality controlled by fastp with default parameters. Then clean reads were separately aligned to the reference genome (Homo sapiens, Version GRCh38.p13) with orientation mode using HISAT2 software. The mapped reads of each sample were assembled by using StringTie^[12]^. Transcript abundances were quantified by RSEM^[13]^ software. The specific parameters in StringTie were "stringtie -p 8 -G ref.gtf -j 1 -c 1 -o sample.primary.gtf -l sample.sort.bam" and "stringtie --merge -p 8 -G ref.gtf -o total.merged.gtf -l merged sample*.gtf". The specific parameters in RSEM was "rsem-prepare-reference --transcript-to-gene-map knowIso.form.txt --bowtie2 ref.exon.fa ref.exon.fa" and "rsem-calculate-expression -p 8 --bowtie2 --paired-end sample.clean.1.fastq.gz sample.clean.2.fastq.gz ref.exon.fa sample".

**Differential expression analysis and functional enrichment**

To identify DEGs (differential expression genes) between two different samples/groups, the gene expression level was calculated based on transcripts per million. Transcript abundances were quantified by RSEM software. DESeq2 was used to detect differentially expressed genes (DEGs) (log2(Fold Change) > 1, *P* value < 0.05). In addition, DEGs were considered significantly enriched in a metabolic pathway at *q* ≤ 0.05 compared with the whole transcriptome background. Functional enrichment analysis was performed using Goatools. Bubble charts were applied to manifest those significantly enriched KEGG pathways (*q* ≤ 0.05). Interaction networks and enriched pathways were constructed using cnetplot^[14]^ function in R language software (v4.0.3).

**Sample size**

On the basis of previous studies^[15]^, we could identify significant microbial, metabolomic, and transcriptomic differences between two groups from individuals with cirrhosis and controls. Therefore, the study including 64 participants with cirrhosis and 59 controls would be adequate to detect meaningful differences.

**References**

1. Chen S, Zhou Y, Chen Y, Gu J. fastp: an ultra-fast all-in-one FASTQ preprocessor. Bioinformatics 2018;34:i884-i890.

2. Magoč T, Salzberg SL. FLASH: fast length adjustment of short reads to improve genome assemblies. Bioinformatics 2011;27:2957-2963.

3. Edgar RC. UPARSE: highly accurate OTU sequences from microbial amplicon reads. Nat Methods 2013;10:996-998.

4. Stackebrandt E , M GB. Taxonomic Note: A Place for DNA-DNA Reassociation and 16S rRNA Sequence Analysis in the Present Species Definition in Bacteriology[J]. International Journal of Systematic Bacteriology 1994;44(4):846-849.

5. Wang Q, Garrity GM, Tiedje JM, Cole JR. Naive Bayesian classifier for rapid assignment of rRNA sequences into the new bacterial taxonomy. Appl Environ Microbiol 2007;73:5261-5267.

6. Schloss PD, Westcott SL, Ryabin T, Hall JR, Hartmann M, Hollister EB, et al. Introducing mothur: open-source, platform-independent, community-supported software for describing and comparing microbial communities. Appl Environ Microbiol 2009;75:7537-7541.

7. Caporaso JG, Kuczynski J, Stombaugh J, Bittinger K, Bushman FD, Costello EK, et al. QIIME allows analysis of high-throughput community sequencing data. Nat Methods 2010;7:335-336.

8. Coker OO, Dai Z, Nie Y, Zhao G, Cao L, Nakatsu G, et al. Mucosal microbiome dysbiosis in gastric carcinogenesis. Gut 2018;67:1024-1032.

9. Zhao K, Wang X, Cha S, Cohn AM, Papandonatos GD, Amato MS, et al. A Multirelational Social Network Analysis of an Online Health Community for Smoking Cessation. J Med Internet Res 2016;18:e233.

10. Housley L, Magana AA, Hsu A, Beaver LM, Wong CP, Stevens JF, et al. Untargeted Metabolomic Screen Reveals Changes in Human Plasma Metabolite Profiles Following Consumption of Fresh Broccoli Sprouts. Mol Nutr Food Res 2018;62:e1700665.

11. Tabassum R, Rämö JT, Ripatti P, Koskela JT, Kurki M, Karjalainen J, et al. Genetic architecture of human plasma lipidome and its link to cardiovascular disease. Nat Commun 2019;10:4329.

12. Pertea M, Pertea GM, Antonescu CM, Chang TC, Mendell JT, Salzberg SL. StringTie enables improved reconstruction of a transcriptome from RNA-seq reads. Nat Biotechnol 2015;33:290-295.

13. Li B, Dewey CN. RSEM: accurate transcript quantification from RNA-Seq data with or without a reference genome. BMC Bioinformatics 2011;12:323.

14. Yu G, Wang LG, Han Y, He QY. clusterProfiler: an R package for comparing biological themes among gene clusters. Omics 2012;16:284-287.

15. Feng J, Zhao F, Sun J, Lin B, Zhao L, Liu Y, et al. Alterations in the gut microbiota and metabolite profiles of thyroid carcinoma patients. Int J Cancer 2019;144:2728-2745.

**Table S1.** Biochemical indices of cirrhotic patients in each group.

| **Biochemical indices** | **Gastric microbiota genomic** | **Serum metabolomic** | | **Gastric mucosa transcriptomic** |  |
| --- | --- | --- | --- | --- | --- |
|  | n=39 | n=22 | n=10 | | |
| ALT, U/L | 29.00±19.53 | 30.9±20.69 | | 25.50±11.69 |  |
| AST, U/L | 31.50±14.03 | 36.67±16.72 | | 32.40±15.19 |  |
| GGT, U/L | 55.83±47.63 | 67.62±46.56 | | 45.10±48.93 |  |
| AKP, U/L | 92.74±72.85 | 60.89±53.92 | | 107.60±78.51 |  |
| TBIL, μmol/L | 18.58±22.46 | 13.03±7.47 | | 38.21±45.30 |  |
| DBIL, μmol/L | 14.80±17.13 | 23.40±16.06 | | 17.15±24.78 |  |
| ALB, g/L | 37.41±5.71 | 37.74±6.63 | | 39.47±5.05 |  |
| TBA, μmol/L | 29.29±29.63 | 23.25±12.46 | | 58.46±62.72 |  |
| Cho, mmol/L | 3.93±1.16 | 4.08±1.25 | | 4.01±0.68 |  |
| HDL, mmol/L | 1.26±0.43 | 1.31±0.46 | | 1.15±0.25 |  |
| LDL, mmol/L | 2.02±0.60 | 2.10±0.57 | | 2.05±0.53 |  |
| TG, mmol/L | 0.85±0.32 | 0.86±0.27 | | 1.30±0.39 |  |
| Glu, mmol/L | 5.64±1.35 | 5.84±1.94 | | 5.61±1.96 |  |
| BUN, mmol/L | 5.23±1.44 | 5.58±1.42 | | 5.64±1.61 |  |
| Cr, μmol/L | 62.71±11.84 | 57.44±7.48 | | 68.80±11.99 |  |
| AMON, μmol/L | 43.42±17.36 | 49.45±14.19 | | 50.00±21.40 |  |

Comparisons between patients with cirrhosis and controls. Data are presented as mean ± standard deviation.

ALT, alanine aminotransferase; AST, aspartate aminotransferase; GGT, gamma-glutamyltransferase; AKP, alkaline phosphatase; TBIL, total bilirubin; DBIL, indirect bilirubin; ALB, albumin; TBA, total bile acid; Cho, serum total cholesterol; HDL, high density lipoprotein; LDL, low density lipoprotein; TG, triglyceride; Glu, glucose; BUN, blood urea nitrogen; Cr, serum creatinine; AMON, blood ammonia.

**Table S2.** Gastric mucosa microbiome alpha diversity index.

| **Alpha diversity index** | **Cirrhosis subjects** | **Control subjects** | **P value** |
| --- | --- | --- | --- |
| Sobs | 263.92±93.05 | 198.33±117.67 | <0.05 |
| Simpson | 0.19±0.24 | 0.49±0.36 | <0.001 |
| Ace | 295.90±100.25 | 237.68±132.40 | <0.05 |
| Chao1 | 298.31±101.83 | 228.92±130.24 | <0.05 |

Comparisons of gastric mucosal flora alpha diversity index between patients with cirrhosis and controls. Data are presented as mean ± standard deviation. P<0.05 on Wilcoxon rank-sum test between patients with cirrhosis and controls.

**Table S3.** PLSDA-VIP table between patients with cirrhosis and controls.

| **Metabolite** | **VIP value** |
| --- | --- |
| Ala Ile Phe Asp | 2.97 |
| Linalool oxide D 3-[apiosyl-(1->6)-glucoside] | 2.75 |
| Schidigerasaponin B1 | 3.27 |
| L-Leucyl-L-Alanine | 3.11 |
| Sterebin D | 3.18 |
| BUTACAINE | 3.16 |
| Guanosine | 3.00 |
| Isoleucyl-Valine | 3.47 |
| Ile Val | 2.73 |
| Cis-5-Tetradecenoylcarnitine | 2.89 |
| L-Hexahydro-3-imino-1,2,4-oxadiazepine-3-carboxylic acid | 2.78 |
| Tyrosyl-Isoleucine | 2.91 |
| Lysyl-Gamma-glutamate | 2.85 |
| 3'-Deaminofusarochromanone | 2.72 |
| Sakuranetin | 3.01 |
| D-Urobilinogen | 2.91 |
| 16-Oxoandrostenediol | 2.83 |
| Endalin | 4.63 |
| Taurochenodeoxycholate-3-sulfate | 3.72 |
| Taurodeoxycholic acid | 2.93 |
| 5beta-CHOLANIC ACID-3alpha, 12alpha-DIOL N-(2-SULPHOETHYL)-AMIDE | 2.80 |
| 3-Oxo-4,6-choladienoic acid | 2.77 |
| METHIONAL | 3.71 |
| 2-Mercaptobenzothiazole | 2.90 |
| Corchorosol A | 5.03 |
| 1-(6Z,9Z,12Z-octadecatrienoyl)-glycero-3-phosphate | 3.21 |
| Neosaxitoxin | 2.96 |
| 2-Hydroxyestrone-4-S-glutathione | 4.60 |
| Jasmonic acid | 2.97 |
| (+/-)14-HDoHE | 2.74 |

Comparisons between patients with cirrhosis and controls. PLSDA-VIP table showed top 30 metabolites with VIP > 1.0 and P value < 0.001.

VIP, variable important in projection.
