# Supplementary figures and images for: A multi-omics study to investigate the progression of the Correa pathway in gastric mucosa in the context of cirrhosis
Source: Gut Pathog. 2023 Sep 26;15:45. doi: 10.1186/s13099-023-00571-y (PMC10521386; doi:10.1186/s13099-023-00571-y)

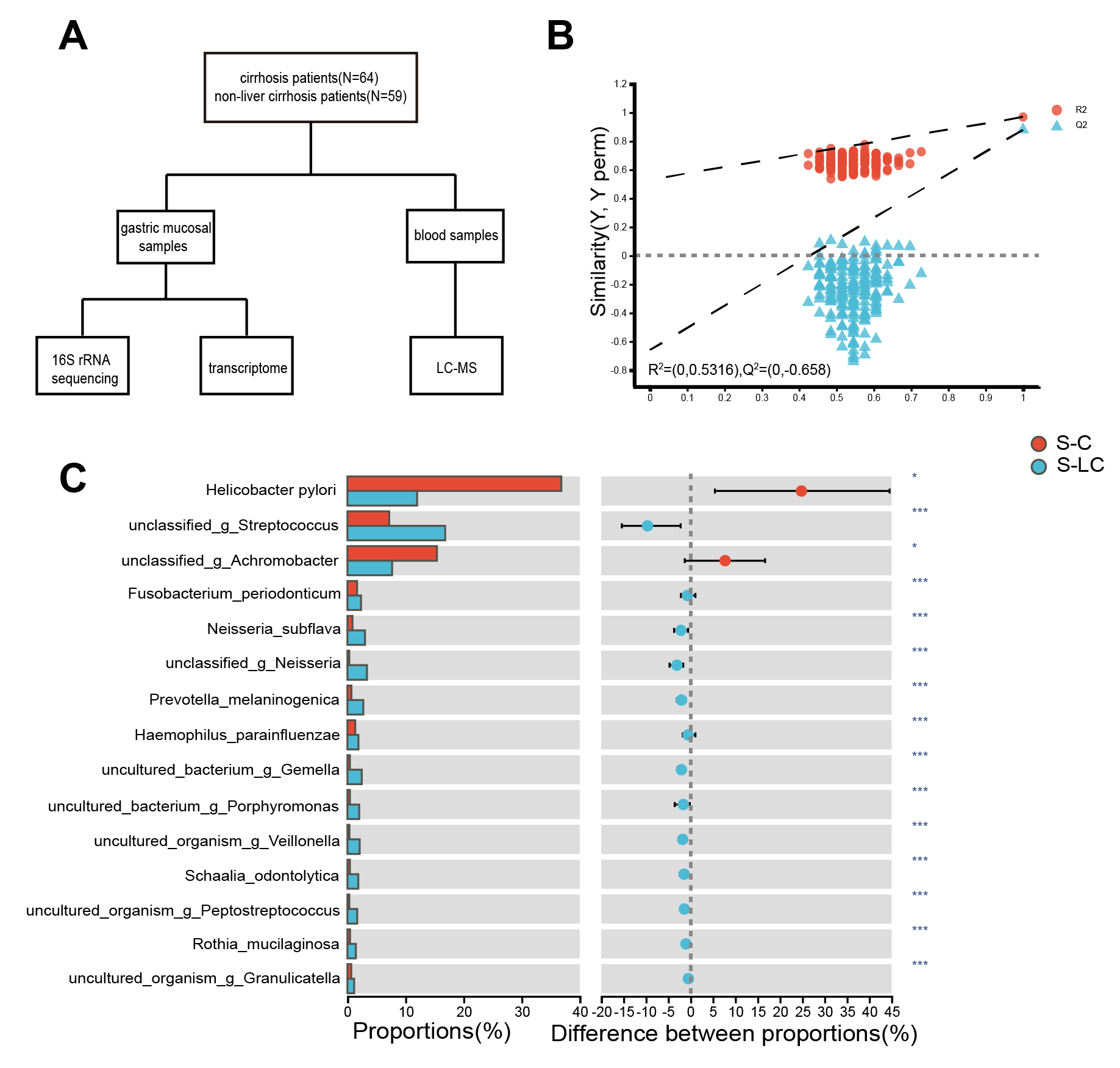

Supplement: Supplementary file 2 — Additional file 2: Figure S1. S-C represents gastric mucosa derived from the control group, and S-LC represents gastric mucosa derived from the liver cirrhosis group. (A) Fig A is the flow chart of the study. (B) Validation of the PLS-DA model using permutation testing. (C) The difference in species composition at the specie level. *p < 0.05, **p < 0.01, and ***p < 0.001. [file 13099_2023_571_MOESM2_ESM.tif]

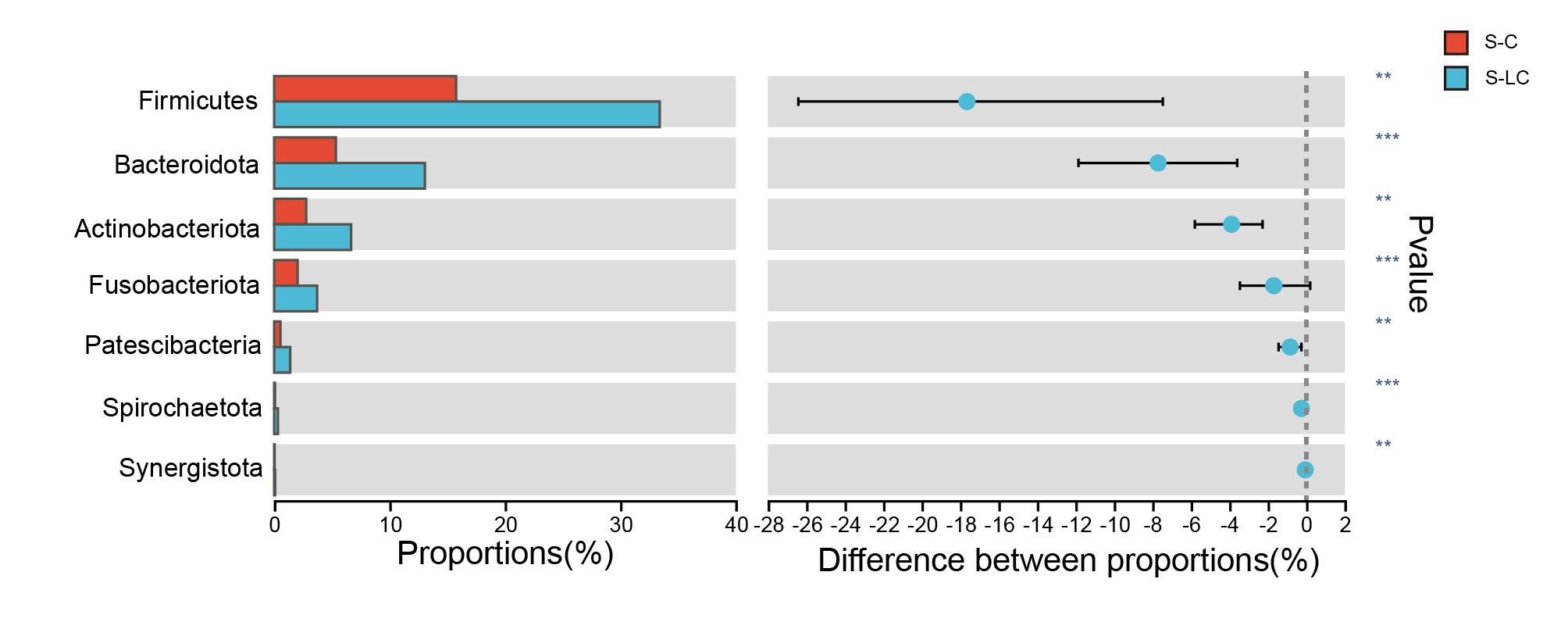

Supplement: Supplementary file 3 — Additional file 3: Figure S2. S-C represents gastric mucosa derived from the control group, and S-LC represents gastric mucosa derived from the treatment group. Wilcoxon rank-sum test bar plot on phylum level. **p < 0.01, and ***p < 0.001. S-C, gastric mucosa in the control group; S-LC, gastric mucosa in patients with cirrhosis. [file 13099_2023_571_MOESM3_ESM.tif]

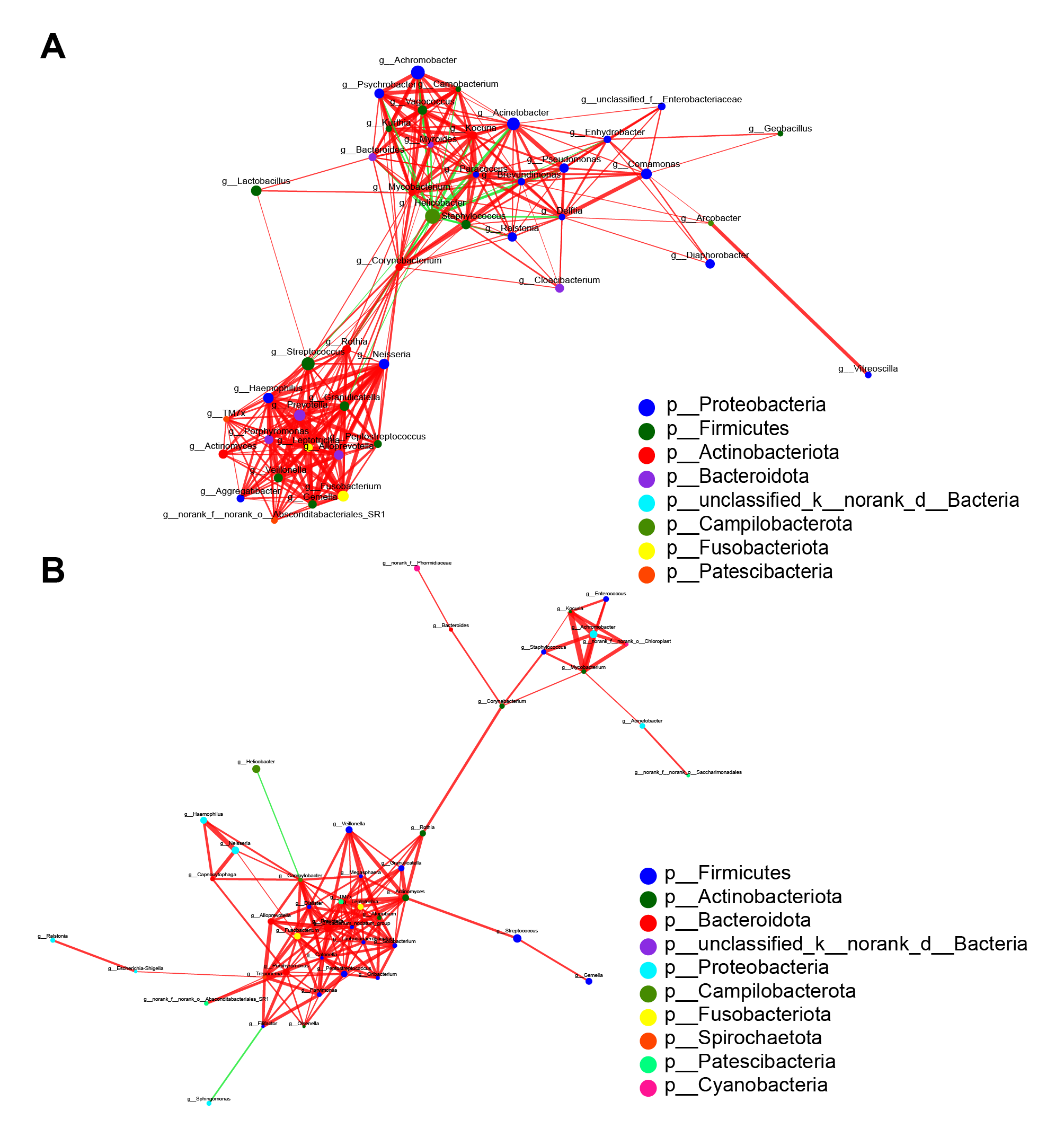

Supplement: Supplementary file 4 — Additional file 4: Figure S3. Network analysis applied to the gastric mucosal microbiota genera network analysis reveals significant interactions. The size of the node is proportional to the species' abundance. Node color corresponds to phylum taxonoic classification. The edge color represents positive (green) and negative (red) correlations, and the edge thickness is equivalent to the correlation values. All Spearman correlations have |r|> 0.5, p < 0.05. Fig A and Fig B represent the control and liver cirrhosis groups, respectively. [file 13099_2023_571_MOESM4_ESM.tif]

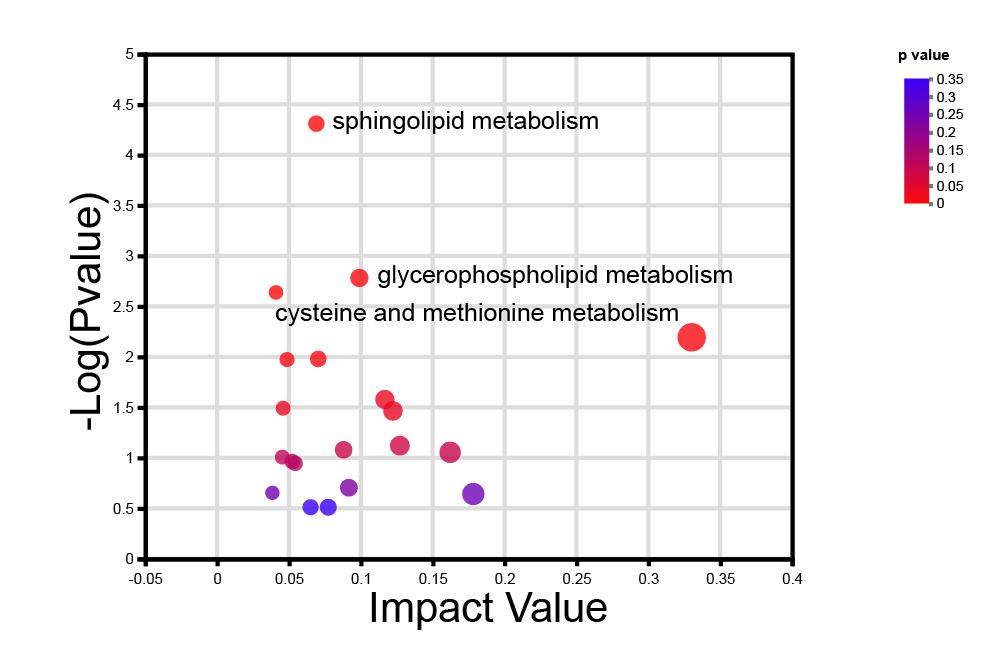

Supplement: Supplementary file 5 — Additional file 5: Figure S4. Metabolite KEGG pathway enrichment map. Each bubble in the graph represents a KEGG Pathway. The X-axis represents the relative importance of metabolites in the pathway in terms of impact value; the Y-axis represents the enrichment significance of metabolite involvement in the pathway—log10(P value). The size of the bubble represents the Impact value. The larger the bubble, the greater the importance of the pathway. KEGG, Kyoto Encyclopedia of Genes and Genomes. [file 13099_2023_571_MOESM5_ESM.tif]

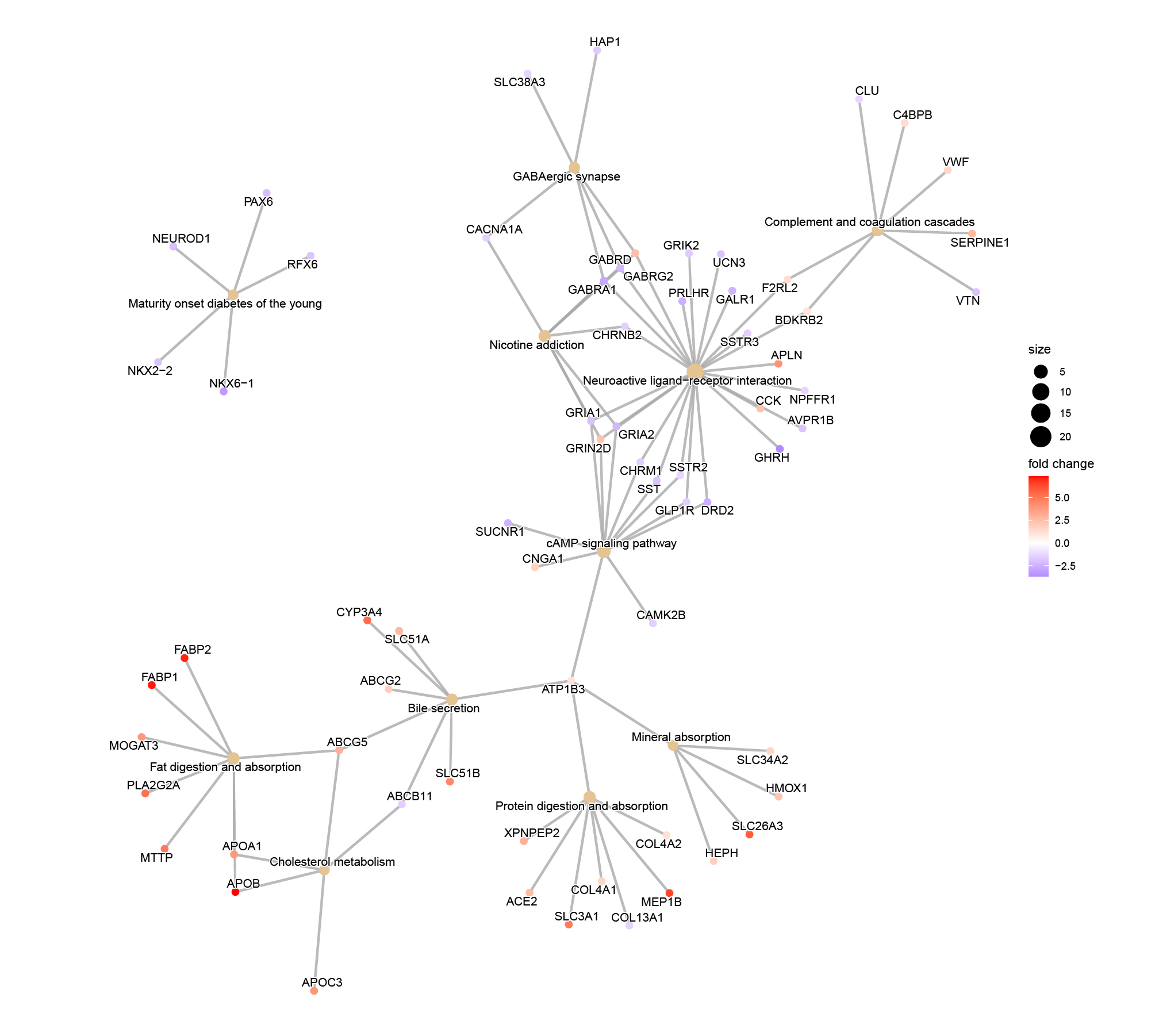

Supplement: Supplementary file 6 — Additional file 6: Figure S5. Cnetplot of top 11 enriched KEGG terms between control and liver cirrhosis group. [file 13099_2023_571_MOESM6_ESM.tif]
